# Structure of Composite Based on Polyheteroarylene Matrix and ZrO_2_ Nanostars Investigated by Quantitative Nanomechanical Mapping

**DOI:** 10.3390/polym9070268

**Published:** 2017-07-06

**Authors:** Maria P. Sokolova, Michael A. Smirnov, Alexander N. Bugrov, Pavel Geydt, Elena N. Popova, Erkki Lahderanta, Valentin M. Svetlichnyi, Alexander M. Toikka

**Affiliations:** 1 Department of Chemical Thermodynamics & Kinetics, Saint Petersburg State University, Universitetsky pr. 26, Peterhof, Saint Petersburg 198504, Russia; Smirnov_Michael@mail.ru (M.A.S.); a.toikka@spbu.ru (A.M.T.); 2 Laboratory of Physics, Lappeenranta University of Technology, Skinnarilankatu 34, 53850 Lappeenranta, Finland; Pavel.Geydt@lut.fi (P.G.); Erkki.Lahderanta@lut.fi (E.L.); 3 Institute of Macromolecular Compounds, Russian Academy of Sciences, Bolshoy pr. 31, Saint Petersburg 199004, Russia; alexander.n.bugrov@gmail.com (A.N.B.); popovaen@hq.macro.ru (E.N.P.); valsvet@hq.macro.ru (V.M.S.); 4 Department of Physical Chemistry, Saint Petersburg Electrotechnical University “LETI”, ul. Professora Popova 5, St. Petersburg 197376, Russian

**Keywords:** polyheteroarylene, zirconia, nanocomposite, polymer structure, quantitative nanomechanical mapping

## Abstract

It is known that structure of the interface between inorganic nanoparticles and polymers significantly influences properties of a polymer–inorganic composite. At the same time, amount of experimental researches on the structure and properties of material near the inorganic-polymer interface is low. In this work, we report for the first time the investigation of nanomechanical properties and maps of adhesion of material near the inorganic-polymer interface for the polyheteroarylene nanocomposites based on semi-crystalline poly[4,4′-bis (4″-aminophenoxy)diphenyl]imide 1,3-bis (3′,4-dicarboxyphenoxy) benzene, modified by ZrO_2_ nanostars. Experiments were conducted using quantitative nanomechanical mapping (QNM) mode of atomic force microscopy (AFM) at the surface areas where holes were formed after falling out of inorganic particles. It was found that adhesion of AFM cantilever to the polymer surface is higher inside the hole than outside. This can be attributed to the presence of polar groups near ZrO_2_ nanoparticle. QNM measurements revealed that polymer matrix has increased rigidity in the vicinity of the nanoparticles. Influence of ZrO_2_ nanoparticles on the structure and thermal properties of semi-crystalline polyheteroarylene matrix was studied with wide-angle X-ray scattering, scanning electron microscopy, and differential scanning calorimetry.

## 1. Introduction

In recent years increasing interest has been focused on the elaboration of polymer–inorganic composites (mixed matrix membranes) as functional hybrid materials, which combine the best properties of both phases: mechanical properties and processability of polymers on the one hand, and electrical, optical, catalytic or transport properties of inorganic materials on the other hand. Polyheteroarylenes, in particular aromatic polyimides and their composites, are promising materials for a wide range of applications due to their superior chemical stability, good mechanical properties, and excellent thermal stability [1]. Introduction of inorganic particles into the polyimides can regulate the physicochemical properties of polymers and attain desirable properties in the material. Recently, it was demonstrated that such composites can be promising as membrane materials for separation of gas and liquid mixtures [2,3,4,5,6], films for optoelectronic fabrications [7,8], and high performance dielectric materials [9].

Different types of inorganic nanofillers are reported as regulators of polyimide properties. It was shown that titania nanoparticles are able to regulate transparency of polyimide films prepared from polymer containing benzimidazole side groups [10]. It is worth noting that successful dispersion of filler in the polymer requires additional dispersants or specific groups attached to the polymer. The authors of [11] describe the preparation of barium titanate/polyimide nanocomposite films using two types of dispersants 2-phosphonobutane-1,2,4-tricarboxylic acid and acrylic-acrylate-amide copolymers. It was shown that surface modification of nanoparticles improves their dispersibility in a polymer matrix and enhances dielectric properties of the films. In the work [12], the end –Si(OH)_3_ groups were attached to the polymer to increase its compatibility with inorganic particles. Additionally, it was shown that inorganic nanoparticles can regulate structure of polyimide membranes, which influence the selectivity of membranes in separation applications [13,14,15]. For example, it was shown that CO_2_/N_2_ and CO_2_/CH_4_ selectivity increase with the addition of ZnO nanoparticles to the polyimide bearing pendent naphthyl groups.

It is generally accepted that introduction of inorganic nanoparticles into organic membrane leads to: decrease of chain mobility near polymer-particle interface [16], change of the free volume of film [4,5], change of the degree of crystallinity, the size of crystallites of polymer and distribution of crystalline phase inside the polymer volume [17,18]. Appearance of new selective diffusion pathways along polymer-inorganic interface affects the selectivity of a membrane. Orientation of polymeric molecules near the surface of carbon nanotubes is important for enhancing mechanical properties of polyimides [19]. It was shown recently [20] by molecular dynamics simulations of polyheteroarylene matrix that polyimide macromolecules form subsurface layers near the surface of single-walled carbon nanotubes. In the case of polyimide prepared from 1,3-bis (3′,4-dicarboxyphenoxy) benzene and 4,4′-bis (4″-aminophenoxy) diphenyl (R-BAPB), the elongation of chains near inorganic particles was observed, while in the case of polymer synthesized with the same dianhydride and 4,4′-bis (4″-aminophenoxy) diphenylsulfone (R-BAPS) chains become compact. Thus, local characteristics and behaviors of polymer in proximity with the surface of inorganic particle are of great practical and theoretical interest.

Atomic force microscopy (AFM) is a versatile method, which can be used for simultaneous mapping of the surface morphology with electrical, mechanical, and adhesive properties of a material with high spatial resolution and with an ability to operate within different ambient conditions [21,22]. For studying of nanomechanical properties PeakForce Quantitative Nanomechanical Mapping (PF-QNM^TM^) technique, which allows the investigation of soft polymeric materials with a spatial resolution of ~50 nm is often applied [23]. PF-QNM has been successfully used for studies of nano-mechanical properties of polymeric materials under different impacts. For example, amyloid fibers at ageing [24], structural evolution, and mechanical properties of a deformed isoprene rubber [25] and the influence of silica on the nanomechanical properties of chitin–silica hybrid film [26,27]. In [28], it was pointed out that PF-QNM is the delicate and simple method to understand the nanoscale structure and to investigate interfacial interactions involved in the compatibilization process in polymer nanocomposites. At the same time, PF-QNM technique has never been used for characterization of nanomechanical properties of polyimide-inorganic composites.

In the present work, we report on our investigation of R-BAPB polyimide/ZrO_2_ nanostars composite using PF-QNM, wide-angle X-ray diffraction (WAXD), scanning electron microscopy (SEM), and differential scanning calorimetry (DSC). The aim was to understand the difference between the properties of polymer matrix near organic/inorganic interface and in the bulk of the composite, which is necessary for further understanding of separation properties of mixed matrix membranes.

## 2. Materials and Methods

### 2.1. Materials

Zirconium (IV) oxychloride octahydrate (98.5%, Neva-Reactive, Saint Petersburg, Russia, CAS: 7699-43-6); sodium acetate trihydrate (99.5%, Neva-Reactive, Saint Petersburg, Russia, CAS: 6131-90-4); *N*-methyl-2-pyrrolidone (97%, Sigma-Aldrich, St. Louis, MO, USA, CAS: 120-94-5); 4,4′-bis (4″-aminophenoxy) biphenyl (97%, TCI, CAS: 13080-85-8); 1,3-bis (3′,4-dicarboxyphenoxy) benzene (OOO «Tech. Chim. Prom.», Yaroslavl, Russia) were used as received without purification.

### 2.2. Synthesis of ZrO_2_ Nanostars

Star-shaped ZrO_2_ nanoparticles were synthesized in hydrothermal conditions from ZrOCl_2_·8H_2_O, by the method described previously in [29]. Zirconium oxychloride (0.805 g) and sodium acetate (0.103 g), i.e., with mole ratio 1:2, were dissolved in 15 mL of distilled water under stirring for 1 h. The obtained solution was transferred to a teflon cell and was hold in autoclave at temperature of 240 °С and pressure of 150 atm for 4 h. The detailed structure of zirconia nanostars prepared by this method was described earlier in [13].

### 2.3. Preparation of Composites

**Figure 1:**
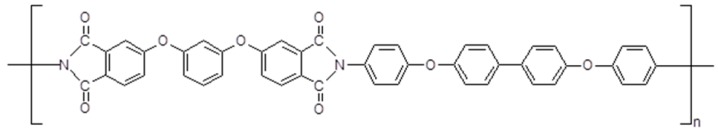
The chemical structure of the polyimide R-BAPB repeating unit.

**Figure 2:**
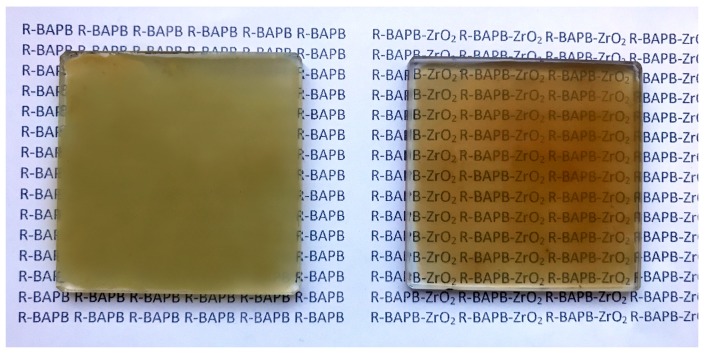
Optical images of pristine R-BAPB (**left**) and composite membrane R-BAPB-ZrO_2_ (**right**).

Two monomers were used for preparation of R-BAPB polyimide: (1,3-bis (3′,4-dicarboxyphenoxy) benzene and 4,4′-bis (4″-aminophenoxy) biphenyl). The chemical structure of the repeating unit of R-BAPB polyimide is given in Figure 1. ZrO_2_ nanostars with concentration 5 wt % based on the weight of a polymer were dispersed in *N*-methyl-2-pyrrolidone. After that, the diamine and dianhydride in a molar ratio of 0.97:1.03 were consistently dissolved in the dispersion of nanoparticles. Formation of polyamic acid was conducted under argon flow during 6 h with continuous stirring. Composites were prepared by casting the solution onto glass plates with subsequent removing of solvent (12 h at 80 °С) and imidization by stepwise thermal treatment: 1 h at 100 °С, 1 h at 150 °С, 1 h at 200 °С, 1 h at 250 °С, 1 h at 280 °С, and 0.5 h at 300 °С. Finally, the obtained films were removed from glass plates for further investigation. Prepared polymer–inorganic composite is denoted in the text as R-BAPB-ZrO_2_. For comparison, the polyimides films without inorganic filler was also prepared and it is denoted as R-BAPB. Thickness of all films was 20 µm. Optical images of prepared films are shown in Figure 2.

### 2.4. Characterization Methods

#### 2.4.1. Microscopic Investigation

Scanning electron microscopy (SEM) micrographs of the films’ surfaces were obtained with a Zeiss Merlin SEM (Carl Zeiss, Oberkochen, Germany). For investigation of cross-sections, the films were frozen in liquid nitrogen and fractured perpendicularly to their surface. Coating with carbon layer was used for preparation of the samples for SEM.

Scanning probe microscope multimode 8 (Bruker, Santa Barbara, CA, USA) operating in PeakForce TUNA^TM^ mode was used for atomic force microscopy (AFM) experiments. Scanning was done in PeakForce QNM mode with feedback adjusted automatically by ScanAsyst program protocol. Major PeakForce parameters were: amplitude 100 nm and frequency 2 kHz. ScanAsyst-Air probe (Bruker, Santa Barbara, CA, USA) with a tip radius of 5 nm and spring constant 0.47 N·m^−1^ was used for accurate topography measurements with setpoint force 2 nN. Then, a considerably stiffer probe Tap525a (Bruker, Santa Barbara, CA, USA) with tip radius ~10 nm, spring constant ~120 N m^−1^, and resonance frequency of 447 kHz was utilized to perform the QNM measurements under a force of ~50 nN that allowed to deform the sample in depth of approximately 1 nm.

#### 2.4.2. Wide-Angle X-ray Diffraction Study

Structure of initial polyimide and composite film was studied by the wide-angle X-ray diffraction (WAXD) with using a D8 DISCOVER diffractometer (Bruker, Rheinstetten, Germany). Scattering angles varied from 5° to 40° with 0.05° step using Cu-K_α_ radiation.

#### 2.4.3. Analysis of Thermal Properties

Differential scanning calorimetry (DSC) was conducted using a DSC 204 F1 (Netzsch, Selb, Germany) differential scanning calorimeter to obtain the glass transition temperature (*T*_g_) of samples. The analysis was conducted under inert atmosphere with samples of approximately 4–5 mg at a scan rate of 10 °C min^−1^ from 20 to 350 °C. Thermobalance TG 209 F1 Libra (Netzsch, Selb, Germany) was used for thermogravimetric analysis (TGA), which was performed under inert atmosphere with samples having a weight of approximately 2–4 mg at a scan rate of 10 °C·min^−1^.

#### 2.4.4. FTIR Spectroscopy Investigation

In this work, the IR Fourier spectrometer Vertex 70 (Bruker, Ettlingen, Germany) and the ATR reflector (Pike Technologies, WI, USA) were used. Zn-Se crystals in the form of prisms with an incidence angle of the radiation on the object θ = 45° were used as ATR elements.

## 3. Results and Discussion

### 3.1. WAXD Data

**Figure 3:**
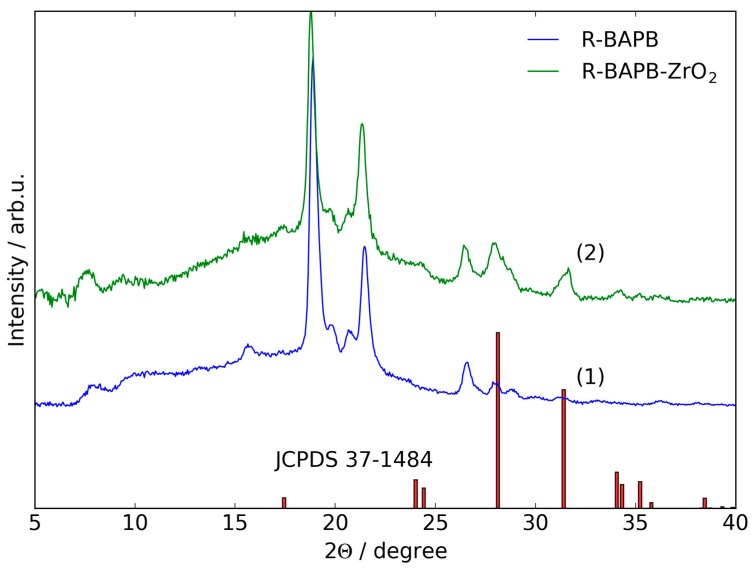
The wide-angle X-ray diffraction (WAXD) patterns of pristine polyimide (R-BAPB) (**1**), its composite with zirconia (R-BAPB-ZrO_2_) (**2**) and reference pattern for monoclinic zirconia (JCPDS 37-1484).

Structure of R-BAPB film and composite (R-BAPB-ZrO_2_) were characterized by WAXD. The diffraction patterns are presented in Figure 3. According to pattern 1 (Figure 3), the initial polyimide represented semi-crystalline structure and positions of the peaks are resemble the spectra reported previously [30]. The sample exhibit two strong reflections at 18.9° and 22°, which correspond to the interplanar distances of *d* = 4.7 and 4.1 Å, respectively, and weak reflections at 2Θ = 8.1°, 10.3°, 13.3°, 15.7°, 19.8°, 20.7°, 26.6°, 28.0°, and 28.9° (*d* = 10.9, 8.6, 6.6, 5.7, 4.5, 4.3, 3.4, 3.2, and 3.1 Å, respectively).

From the diffraction pattern of composite film (Figure 3, pattern 2) it is seen that the main reflections are located at the same positions as the peaks of initial polymer. However, their intensity is slightly reduced in comparison with the pure polymer matrix. In addition, strong reflections with 2Θ = 24.1°, 28.1°, 31.5°, 34.2°, and 35.2° (*d* = 3.7, 3.2, 2.8, 2.6, and 2.5 Å) appear, which corresponds to the presence of ZrO_2_ nanostars with monoclinic singony. Positions of the peaks for ZrO_2_ from the JCPDS card No. 37-1484 are shown in Figure 3 for comparison with an experimental diffraction pattern. The presented data confirm the chemical structure of initial polyimide and qualitative chemical composition of its composite with zirconia.

### 3.2. Investigation of Morphology with Electron Microscopy

**Figure 4:**
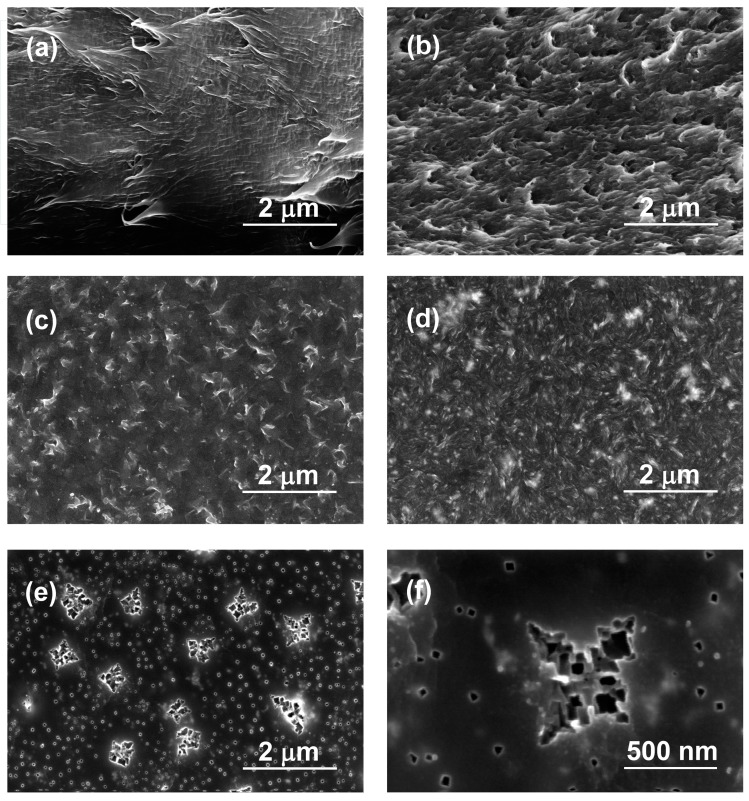
Scanning electron microscopy (SEM) images of pristine polyimide film R-BAPB (**a**,**c**) and composite film with zirconia (**b**,**d**,**e**,**f**). Cross-section morphology (**a**,**b**), images of upper side (**c**,**d**) and bottom side (**e**,**f**) of films.

SEM results of cross-sections and surfaces of initial R-BAPB and its composite with ZrO_2_ are presented in Figure 4. A lot of fracture lines are clearly visible on the image of cross-section of initial polyimide (Figure 4a). This is connected to the semi-crystalline nature of the polymer. Addition of zirconia nanoparticles leads to the significant changes in the morphology of cross-section (see Figure 4b). Roughness of cross-section significantly increases, which can relate to the presence of nanoparticles in the polymer matrix. It can be seen from Figure 4c that the upper surface of pure R-BAPB film is composed from uniformly distributed crystalline flake-like domains with sizes about 400 nm. Introduction of zirconia nanoparticles decreases the sizes of crystalline domains to 100–200 nm (Figure 4d). This is connected to possible ability of zirconia to act as crystallization center for the polymer, which leads to formation of increased amount of crystallites with reduced size.

Thus, SEM images of composite membranes based on R-BAPB and ZrO_2_ nanostars show excellent homogeneity of the prepared films, where agglomerated inorganic particles are not visible. It can be proposed that, due to possible ability of ZrO_2_ particles to act as crystallization centers, growing polymer crystallites around ZrO_2_ particles push them apart from each other. It is possible that the interaction between surface –OH groups of ZrO_2_ and oxygen atoms of carbonyl groups of imide cycles of polymer via hydrogen bounding leads to the orientation of polymer chains and their denser packing in the vicinity of the nanoparticle than in the bulk of the polymer. This interaction will be discussed in the Section 3.3. As a result, the uniform distribution of isolated nanoparticles is achieved.

Presented images confirm that the preparation method based on introduction of nanoparticles before the synthesis of polyamic acid leads to strongly uniform distribution of filler in the composite.

Figure 4e,f show morphology of bottom side of the composite film with different magnifications. It can be seen that surface of the film is smooth and uniformly distributed nanoparticle are clearly visible. Along with the small nanostars with diameters about 50 nm, the bigger ones with diameters 500 nm are seen. They appear only on the bottom side of the composite film, not in the cross-section or upper surface, which can be explained by sedimentation of bigger particles during solvent evaporation from composite (R-BAPB-ZrO_2_). At the same time, it must be noticed that the SEM method is unable to provide a definite answer to the question if visible stars are inorganic particles or only their imprint on the surface of polymer. However, appearance of such objects makes it possible to investigate the structure and properties of polymeric matrix near inorganic nanoparticles. This was performed with AFM measurements of the bottom side of the composite membrane.

### 3.3. FT-IR Spectroscopy

**Figure 5:**
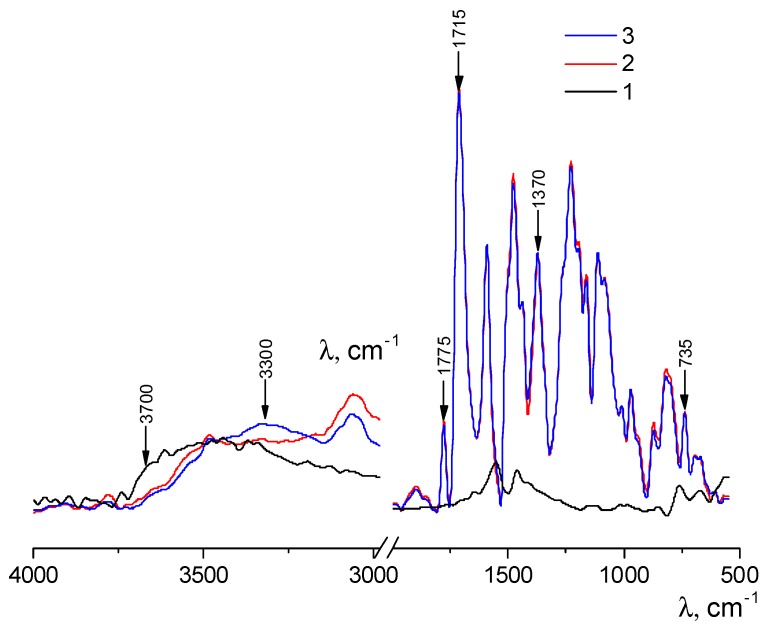
FT-IR spectra of zirconia nanostars (**1**), pristine polyimide (**2**) and composite film with ZrO_2_ (**3**).

The chemical structure of pristine polyimide R-BAPB and composite film was investigated with FT-IR spectroscopy (Figure 5). Comparison of FT-IR spectra of zirconia nanostars (Figure 5a) and composite film (Figure 5c) demonstrates that the incorporation of nanostars to the polyimide leads to the decrease in the integrated intensity of the band at 3700 cm^−1^ corresponding to free –OH groups on the filler surface and the increase of the intensity of the band in the region of 3300 cm^−1^, which is attributed to the associated hydroxyl groups (Figure 5a,c). These results can be attributed to the changing of the network of hydrogen bonds in a composite in comparison with the initial polymer. FT-IR spectra of initial polyimide and composite film with ZrO_2_ (Figure 5b,c) demonstrate typical bands for the imide cycle: at 1775 cm^−1^ (C=O asymmetric stretching), at 1715–1720 cm^−1^ (C=O symmetric stretching) and at 735 cm^−1^ (C=O banding), with the C–N stretching peak at 1370 cm^−1^.

### 3.4. AFM Results

**Figure 6:**
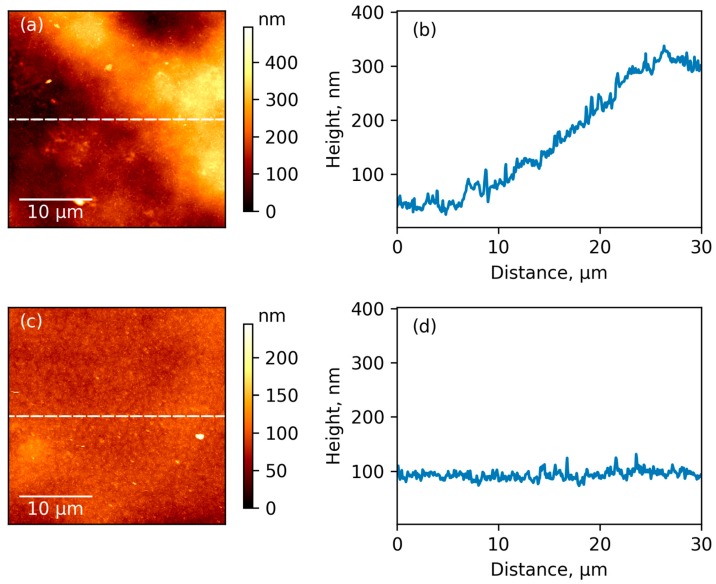
Surface topography of upper side of R-BAPB film (**a**), composite film with zirconia (**c**), and profile curves (**b**,**d**).

Comparison between typical images of surface topography of pure R-BAPB film and its composite membrane with ZrO_2_ is given in Figure 6a,c, respectively. The pristine polymer demonstrates significantly rough surface with considerable difference in height for the neighboring parts of the surface (see profiles curves in Figure 6b,d). Amplitude of topography profile curves is about 350 and 30 nm for initial polymer and composite, respectively. Higher amplitude for pure polyimide can be attributed to the formation of bigger crystallites, which pack in large supramolecular formations during the preparation of the film from the pristine polymer. The root mean squared roughness (R_q_), which was averaged from the values obtained for different parts of surface with sizes 5 μm × 5 μm were 14 ± 2 and 8 ± 0.2 nm for initial polyimide and composite film, respectively. Their difference is emphasized by comparing surface topography profiles which are presented in Figure 6b,d. We suggested that lower roughness and amplitude of profile curve for composite relates to the possible ability of ZrO_2_ nanoparticles to act as crystallization centers for polymer. This leads to an increased amount of crystallites, but with reduced sizes. This is in agreement with our SEM results (Figure 4a,b). AFM pictures also demonstrate uniformity of distribution of inorganic particles inside the composite. The reduced roughness due to changes in crystalline structure of composite leads to an increasing glossiness in polymer film with incorporation of inorganic nanoparticles (Figure 2).

**Figure 7:**
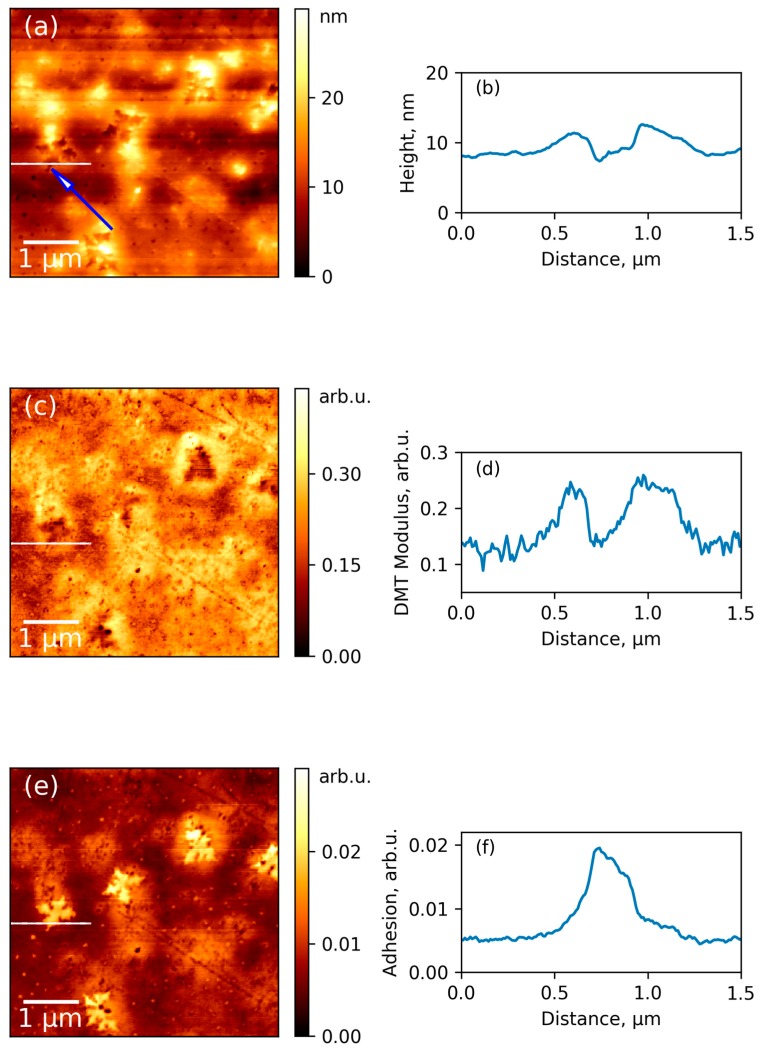
Surface topography of bottom side of composite (**a**), map of elastic modulus (**c**), and map of adhesion (**e**); (**b**,**d**,**f**) show profiles for these corresponding channels taken at the selected region of the surface with an imprint from zirconia nanoparticle (shown with white line segment).

The abovementioned AFM images were collected from the upper surface of the film, which was in contact with air during preparation of sample. Further investigation of the bottom side of the film provided additional information about changes of polymer structure near the organic/inorganic interface. The simultaneously captured surface topography, mechanical stiffness, and adhesiveness of the same area of the bottom surface of composite film are presented in Figure 7a,c,e, respectively. These results were also obtained in the PeakForce QNM mode. As it is seen from the topography map, during removal of the film from substrate, some star-shaped ZrO_2_ particles, which were deposited on the bottom surface of the film, fall out of polymer matrix. The imprint of the star marked with blue arrow in Figure 7a was chosen for measurements of profiles and discussion of results due to the flatness of the surrounding surface. A smooth and flat surface allows to minimize errors, which can arise from different contact area between probe and sample in various points of the rough surface. Figure 7b demonstrates that the depth of the hole remaining in the polymer after removing of the ZrO_2_ nanoparticles is ~4 nm. Therefore, it can be concluded that ZrO_2_ nanostars are composed of flat crystallites with a thickness of ~4 nm and a width of ~450 nm.

Figure 7c,d show the map of elastic modulus and its profile on the same region of surface as presented in Figure 7a,b. It is clearly seen that the sample demonstrates increased stiffness near the position of inorganic particle. This gives evidence for more dense packing of polymeric chains near the nanoparticle than in other parts of the sample. The selected nanoparticle influence on the elastic modulus in the lateral direction is a distance of approximately 500 nm. The same qualitative results can be seen for other regions of the surface. This data is in the agreement with other theoretical results, which demonstrates the ability of R-BAPB chains to elongate near the surface of inorganic particle (carbon nanotube) [20], which was considered as a pre-crystallization stage. Experimental results for composites of polyimides also demonstrate an increase of Young’s modulus of polymeric films with introduction of inorganic nanoparticles [13,31]. At the same time, only nanomechanical measurements with AFM can provide direct experimental evidence for the increasing of local stiffness of polymer matrix near organic/inorganic interface.

Additional insight into the local structure of composites can be obtained from the map of adhesion (Figure 7e). It is seen that the bottom part of the imprint is significantly more adhesive than the surrounding polymeric surface. The profile in the Figure 7f demonstrates that the border of the adhesive region coincides perfectly with the walls of the imprint. It can be suggested that the adhesive properties of this area are connected with high local concentration of polar chemical groups on the bottom surface of the filler imprint.

**Figure 8:**
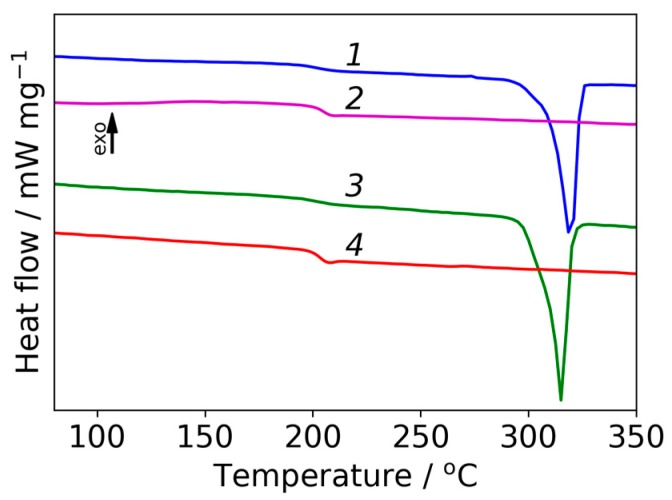
DSC curves of initial polyimide film: first scan (**1**), second scan (**2**) and of composite film with ZrO_2_ nanostars: first scan (**3**), second scan (**4**).

Prepared samples were also investigated with DSC (Figure 8). It was found that glass-transition temperature (*T*_g_) was 203–205 °C for both samples. Peaks corresponding to melting of crystalline phase are clearly visible in the range 315–319 °C, which confirms the semi-crystalline nature of prepared films as it was observed by WAXD. The thermal stability of the prepared samples was evaluated by TGA. The TGA curves indicate that solvent has been successfully eliminated from polyimide film and also from composite film with ZrO_2_ because there is no weight loss below 100 °C. Both TGA curves show one region of weight loss, which correspond to the decomposition of the polymer backbone. The step of weight was 500 °C for pristine polyimide and 480 °C for composite film with ZrO_2_, which agrees with data published in for R-BAPB [30]. This demonstrates a high thermal stability of the prepared samples.

## 4. Conclusions

Nanomechanical properties of composite of semi-crystalline poly [4,4′-bis (4″-aminophenoxy) diphenyl]imide 1,3-bis (3′,4-dicarboxyphenoxy) benzene (R-BAPB) with zirconia nanostars were studied using the quantitative nanomechanical mapping (QNM) mode of atomic force microscopy. Experimental evidence of increased rigidity of polymer near the organic/inorganic interface was obtained for the first time. The distance on which nanoparticle gives influence on the mechanical properties of polymeric matrix is in the range of hundreds of nanometers, which was observed by nanomechanical mapping. Comparison of adhesive properties between surface in the imprint of nanoparticle and upper surface of the membrane material revealed the existence of adhesive polar groups in the surface, which was formed at the contact with zirconia. Investigation of surfaces and cross-sections of pure pristine polyimide (R-BAPB) and composite (R-BAPB-ZrO_2_) films shows that addition of zirconia nanoparticles before the formation polyamic acid can achieve a highly uniform distribution of inorganic filler inside the polymer matrix. Introduction of zirconia nanoparticles significantly decreases the roughness of film’s surface. This can relate to the ability of zirconia nanoparticles to act as crystallization centers during the thermal treatment of a film. As a result, the number of crystalline domains in the composite increases, while their size decreases. Therefore, an ordering effect of inorganic nanoparticles toward the polymer matrix was observed with advanced AFM technique. These results are of interest for using polyheteroarilene films as membrane materials in separation technology.
